# The Effect of Active Learning Methodologies on the Teaching of Pharmaceutical Care in a Brazilian Pharmacy Faculty

**DOI:** 10.1371/journal.pone.0123141

**Published:** 2015-05-13

**Authors:** Alessandra R. Mesquita, Werlissandra M. Souza, Thays C. Boaventura, Izadora M. C. Barros, Angelo R. Antoniolli, Wellington B. Silva, Divaldo P. Lyra Júnior

**Affiliations:** Department of Pharmacy, Federal University of Sergipe, São Cristovão, Sergipe, Brazil; University of Westminster, UNITED KINGDOM

## Abstract

**Background:**

In recent years, pharmacists have been involved in expanded patient care responsibilities, for example patient counseling in self-medication, medication review and pharmaceutical care, which require graduates to develop the necessary competences. Consequently, reorientation of pharmacy education has become necessary. As such, active learning strategies have been introduced into classrooms to increase problem-solving and critical thinking skills of students. The objective of this study was to evaluate the performance and perceptions of competency of students in a new pharmaceutical care course that uses active learning methodologies.

**Methods:**

This pharmaceutical care course was conducted in the first semester of 2014, in the Federal University of Sergipe. In the pharmaceutical care course, active learning methods were used, consisting of dialogic classroom expository, simulation and case studies. Student learning was evaluated using classroom tests and instruments that evaluated the perception of competency in pharmaceutical care practice. Furthermore, students' satisfaction with the course was evaluated.

**Results:**

Thirty-three students completed the four evaluations used in the course (i.e., a discursive written exam, seminars, OSCE, and virtual patient); 25 were female (75.75%), and the median age was 23.43 (SD 2.82) years. The overall mean of student scores, in all evaluation methods was 7.97 (SD 0.59) on a scale of 0 to 10 points, and student performance on the virtual patient method was statistically superior to other methods. With respect to the perception of competency in pharmaceutical care practice, a comparison of pre- and post-test scores revealed statistically significant improvement for all evaluated competences. At the end of the semester, the students presented positive opinions of the pharmaceutical care course.

**Conclusions:**

The results suggest that an active learning course can enhance the learning of pharmaceutical care competences. In future studies it will be necessary to compare active learning to traditional methods.

## Introduction

In recent years, health systems in both the developed and developing world have faced the challenges of financial constraints, a high prevalence of non-communicable diseases, and an increase in problems related to pharmacotherapy and morbidity and mortality associated with drug use [1,2]. These changes in health care have had a great impact on the field of pharmaceutical practice and on pharmacy as a profession [3]. According to the World Health Organization (WHO), pharmacists need to be more involved in solving health care problems, and several studies have demonstrated that this profession has a vital role to play in drug therapy management, which may enhance overall patient health outcomes [4–7].

In response to economic and societal needs, the pharmacy profession has adopted pharmaceutical care as a philosophy of practice. Pharmaceutical care is defined as the responsible provision of drug therapy for the purpose of achieving definite outcomes that improve a patient’s quality of life [8]. This philosophy of practice is understood as the pharmacists’ commitment to obtain the maximum benefit from the pharmacological treatments of the patients, being therefore responsible for the monitoring of their pharmacotherapy [8]. As the profession has moved from a product orientation (dispensing medications) to a patient focus, clinical training requirements have expanded [9].

Since the introduction of this concept, numerous studies have been conducted to evaluate the pharmacist’s capacity to positively influence the results of drug therapy through pharmaceutical care [10–12]. In this role, pharmacists will be involved in expanded patient care responsibilities, which require graduates to possess adequate knowledge, enhanced communication skills, greater problem-solving capabilities, effective critical-thinking abilities, and sound decision making skills [13, 14]. Consequently, a reorientation of pharmacy education is necessary. Curriculum modifications and various instructional strategies will have to be considered to facilitate learning outcomes, in terms of the knowledge and skills needed to practice pharmaceutical care [13, 15].

In this context, active learning strategies have been introduced into classrooms to increase problem-solving and critical-thinking skills of students in a more student-centered environment [16]. Examples of active learning instructional strategies include evaluating case studies, class discussions, project-based learning, problem-based learning, simulation (role playing, simulated patient, and virtual patient), game-based learning, and building concept maps. In the United States, the Accreditation Council for Pharmacy Education (ACPE) and the Center for the Advancement of Pharmacy Education (CAPE) emphasize a patient-centered care practice and note that the curriculum must produce graduates with mature critical thinking and problem-solving skills, and the ability to self-direct their learning [17, 18].

ACPE supports the use of active learning techniques in every phase of pharmacy students’ education and in pharmacists’ continuing professional development [17]. In consequence, active learning methodologies have been used with success in several countries of the world. A systematic review carried by authors, with the purpose to analyze the published studies about the teaching of pharmaceutical care found 21 studies, in countries as Canada, United States, United Kingdom, Taiwan and New Zeeland. The teaching methods more used were simulation (simulated patient and role play), problem-based learning and case studies.

In Brazil, the National Guidelines for Undergraduate Education in Pharmacy recommend professionals' training to consist of a generalist, humanist education, which develops critical thinking skills with respect to health care. In addition, the recommendations target theoretical and practical training in patient care [19]. In 2013, the Brazilian Association of Pharmaceutical and Biochemical Education (Abenfarbio) published a book aiming to guide and encourage the application of active learning methodologies in pharmaceutical education [20]. However, in the same systematic review cited, was found only one study of the use of active learning for pharmaceutical care education in Brazil.

Independent of the teaching method chosen, quality in higher education should be measured in terms of what students know, understand, and can do at the end of the curriculum [21]. To meet these criteria, our objective was to redesign the pharmaceutical care course at the Federal University of Sergipe to include new teaching methods that provide students with basic knowledge and the skills needed to provide pharmaceutical care. The pharmaceutical care course was introduced at our institution in 2005 and is a 16-week long mandatory four-credit course, taught during the fifth year of the undergraduate program.

Specific objectives of this research study were: (1) to evaluate the performance of students in the course, (2) to measure students’ perceptions of their pharmaceutical care competencies before and after the course, and (3) to determine students’ perception of the value of the course to professional practice.

## Methods

### Course Design

The pharmaceutical care course was redesigned in 2013, by one teacher and two other course developers, with the objective of making it more learner-centered. The teacher has a PhD in pharmaceutical care, and ten years of experience in teaching and practice; the two developers were PhD students in pharmacy. One of the two developers was a hospital pharmacist for two years and the teacher of a pharmaceutical services course for one year, while the other had been a community pharmacist for three years and a preceptor in a pharmaceutical care clerkship. All three individuals also served as course instructors.

Before initiating changes in the course, a focus group of expert teachers was convened to support the development of the course syllabus. The focus group aimed to (1) identify the competencies that are necessary for the practice of pharmaceutical care, and (2) develop specific learning activities and teaching methods that would foster these competencies. Furthermore, the three developers held meetings to share ideas about the needs, objectives, teaching strategies, and content of the course.

After the contents and teaching strategies were determined, the new pharmaceutical care course was implemented in 2013 to evaluate the students’ experiences with the course and obtain information about their preferences regarding the learner-centered approach. In this step, the vast majority of the students reacted positively to the innovative course. Changes to the course were implemented based on feedback received from these previous classes.

The overall goals of the course included obtaining an understanding of the pharmaceutical care philosophy and developing the competencies required (i.e., knowledge and skills) to provide pharmaceutical care. The pharmaceutical care model embraced by this course was based on the Strand and Hepler philosophy of the processes of care [8]. Briefly, the students in the course were expected to assess their patients’ drug-related needs and manage those needs through appropriate interventions, including education, monitoring, and follow-up care. The learning objectives of the course, in accordance with Bloom’s taxonomy [22], are presented in Table 1.

**Table 1:** Learning objectives of the pharmaceutical care course.

◾ Identify medicines responsible for increased morbidity and mortality
◾ Explain how the pharmacist can act as a health professional in order to reduce morbidity and mortality related to medicines
◾ Explain the emergence of clinical services in pharmacy
◾ Define pharmaceutical care
◾ Describe the importance of pharmaceutical care to patients and to pharmacists
◾ Explain who are eligible patients to participate in pharmaceutical care
◾ Define macrocomponents of pharmaceutical care
◾ Perform a search for information in databases
◾ Measure clinical parameters (blood pressure, capillary glicemy)
◾ Demonstrate drug administration
◾ Communicate with patients, caregivers, and healthcare practitioners
◾ Collect data using a systematic approach
◾ Identify drug-related needs of patients
◾ Identify drug-related problems
◾ Differentiate real and potential drug-related problems
◾ Prioritize the resolution of drug-related problems
◾ Solve drug-related problems
◾ Develop a pharmaceutical care plan
◾ Document a pharmaceutical care plan
◾ Formulate educational intervention
◾ Provide educational interventions for patients
◾ Refer patients to other health care professionals

### Teaching methods used

In the pharmaceutical care course, students were required to participate in a variety of direct and indirect patient care activities in order to develop skills and knowledge adequate to ensure effective, safe, and convenient drug therapy for patients. Table 2 summarizes the content of the pharmaceutical care course, and the learning strategies used.

**Table 2:** Specific lessons in the Pharmaceutical Care course and their corresponding teaching methods and learning strategies.

Contents	Teaching Method or Learning Strategy	Description of method
Drug-related morbidity and mortality; Pharmaceutical care: historical and conceptual aspects	Dialogic classroom expository	Expository lessons, promoting dialog between teachers and students. The construction of knowledge occurs through the exchange of information, the asking of questions, and discussions about and reflections on reality. Students, because they bring their knowledge and life experience to the classroom, play a major part in the teaching and learning that takes place.
Drug administration; Measurement of clinical parameters	Role play	In the role-playing exercises, the pharmacy students must initiate patient-pharmacist interactions, assess clinical parameters, offer counseling concerning medication use, and/or to invite the patient to use pharmaceutical care services. In this lesson the patient role is played by another pharmacy student. At the end of the scene the roles are reversed. The roles were distributed, allowing each student 5–10 minutes to review his/her role and ask the instructors for clarifications of the questions.
Communication skills; Establishment of the therapeutic relationship; Invitation to use pharmaceutical care services	Simulated Patient	A postgraduate pharmacy student trained to play the role of a patient presents a standardized scenario. The simulated patient interacts with the pharmacy student and the student works to establish a therapeutic relationship and conducts an initial evaluation.
Implementation of pharmaceutical care service	Lecture	Presentation of topics by an invited professional pharmacist expert
Drug information resources; Determination of desired clinical and pharmacotherapeutic outcomes; Identifying, preventing, and solving drug-related problems; Prioritizing drug-related problems and establishing measurable endpoints; Care plan development	Case studies	Through a series of discussions of cases, students are expected to be able to search for evidence-based information about the health problems mentioned and related pharmacotherapies. In addition, students will acquire and/or reinforce their skills in determining whether a patient's undesirable signs/symptoms are related to drug therapy, and if so, to determine how these symptoms are related to the drug therapy and how the drug therapy problem should be solved.
Initial Assessment of a patient: Determine who your patient is as an individual by learning about the reason for the encounter, the patient's demographic characteristics and experiences with medications, and other relevant clinical information; Identifying, preventing, and solving drug-related problems; Prioritizing drug-related problems and establishing measurable endpoints; Care plan development	Virtual Patient	Virtual clinical cases which the students must perform since assessment of patients until development of pharmacotherapy care plan, written communication with healthcare professionals and development of educational interventions. Besides to training of students to interactively document the process of pharmaceutical care.

### Participants

All of the students who were enrolled in the pharmaceutical care course of the undergraduate pharmacy program at the Federal University of Sergipe in the first semester of 2014 were asked to participate in the study. The students were advised of the goals of the study and the fact that the collected data would be confidential. Information on the participants’ age, gender, and year in the undergraduate pharmacy program was collected. Only students who did not consent to participate were excluded from the study.

### Evaluation

To evaluate performance, during the course students were subjected to several university-required evaluations of knowledge, all based on the learning objectives established for the pharmaceutical care course. The first evaluation consisted of a discursive written exam of five questions, in which students were required to explain how the pharmacist can act as a health professional in order to reduce morbidity and mortality related to drugs, to describe the concept of pharmaceutical care, to explain which patients are eligible to participate in pharmaceutical care services, to identify drug-related needs of patients, to identify drug-related problems, and to propose pharmacotherapeutic interventions.

Students were also evaluated via seminars based on the resolution of clinical cases. This evaluation method focused on identifying the drug-related needs of patients, identifying and solving drug-related problems, prioritizing drug-related problems, and establishing measurable endpoints and care plan development. Furthermore, the Objective Structured Clinical Examination (OSCE) was used, with the objective of assessing the students’ competency in measurement of clinical parameters, patient counseling regarding drug administration, communication with patients, and invitation to use pharmaceutical care services. It is worth noting that in the OSCE, students are also asked to perform self-assessment of their performance.

Finally, students were evaluated using virtual patient software. This software was developed in 2010 by the Laboratory of Education and Research in Social Pharmacy and the Laboratory of Computer Science of the Federal University of Sergipe, Brazil, with aim of improving the competencies of students in pharmaceutical care practice [23]. The learning objectives contemplated in this activity consisted of searching drug information databases; collecting and evaluating data using a systematic approach; identifying drug-related needs of patients; identifying, classifying, and solving drug-related problems; prioritizing drug-related problems and establishing measurable endpoints; and care plan development. The latter consisted of documenting the care plan, proposing pharmacotherapeutic interventions, referring the patient to healthcare professionals via written communications, and formulating and providing educational interventions.

Students received scores from 0 to 10 on each evaluation method. At the end of the semester, students were asked to answer four questions about the evaluation methods: (1) In this type of method I feel a lot of pressure to perform well, (2) This method is useful in verifying my learning, (3) The criteria for correctness of the assessments were appropriate, (4) The assigned grade reflects my learning. The questions were answered using a Likert scale ranging from 1 (“strongly disagree”) to 5 (“strongly agree”).

In addition to the university-required evaluations, students completed two instruments pertaining to the course activities. One of these assessed the perception of competence in pharmaceutical care practice, and the other evaluated student satisfaction with the course.

Perception of competence in pharmaceutical care practice: A quasi-experimental, two-group pretest and posttest design was utilized to evaluate students’ perception of their preparedness to perform pharmaceutical care competencies. The survey instrument was designed by the authors, based on other instruments reported in the literature [24–26]. Furthermore it was generated using the steps of pharmaceutical care process proposed by Cipolle, Strand, and Morley [27]. The instrument was successfully tested on a small group of students during the second semester of 2013. Feedback from the pilot group resulted in minor wording changes to improve readability and clarity. The questionnaire collects respondent characteristics, and presents 25 items related to competence and practical skills in the pharmaceutical care process. The questions are evaluated using a Likert scale ranging from 1 (“strongly disagree”) to 5 (“strongly agree”). On conclusion of the pharmaceutical care course, a post-test using the same items was administered to students.

Evaluation of satisfaction with the course: A student opinion survey was conducted in the final week of the semester that assessed general aspects of the course, including whether the teacher had been able to provide them with the intended types of experience, and their own performance on the course. Survey items were answered using a five-point Likert scale ranging from 1 (“strongly disagree”) to 5 (“strongly agree”). The instrument used was developed by the authors based on the related literature [25, 28–31]. The students were also asked whether they preferred that courses be taught using active learning methods. Preferences were assessed via two dichotomous (yes or no) questions.

### Data analysis and ethical aspects

Statistical analysis was performed using the Statistical Package for the Social Sciences (SPSS), version 17. Descriptive statistics were calculated for the questionnaire items, consisting of means, standard deviations (SDs), and percentages of agreement or disagreement with each item. The Shapiro-Wilk test of normality indicated that the data were not normally distributed. Thus, a Wilcoxon sign-ranked test was performed to compare overall pre-test and post-test scores on the instrument measuring the perception of competence in pharmaceutical care practice. The Kruskal-Wallis test was used to examine differences among the students’ performance in the evaluation methods used in the course, and their preference for teaching method.

The level of significance for all statistical analyses was set at *p* < 0.05. All of the responses were anonymous.

The study was approved by the Human Research Ethics Committee of the Federal University of Sergipe (CAAE: 26429413.8.0000.5546). The participants provide their written informed consent to participate in this study and the Human Research Ethics Committee of the Federal University of Sergipe approve this consent procedure.

## Results

Thirty-four students were enrolled in the course. A different number of students completed each of the assessment/evaluation components due to a time gap between the assessments. Thirty-three students (97%) completed the four evaluations used in the course (discursive written exam, seminars, OSCE and virtual patient). The median age was 23.43 years (SD 2.82 years) and 25 were female (75.75%). All students were in the fourth year of the undergraduate pharmacy program.

For this pharmaceutical care course, no students failed. The overall average grade of students was 7.97 (± 0.59) on a scale of 0 to 10 points. The students’ average scores for each examination are presented in Table 3. Student performance on the virtual patient was statistically superior to other methods, with an average score of 9.40 (± 0.41). Moreover, according to the students, the evaluation through virtual patient is useful in verifying learning, and with this method students felt less pressure to perform well. The assessment of students for items “the criteria for correctness of the assessments were appropriate” and “the assigned grade reflects my learning” were positive. However no significant differences between the assessment methods were observed.

**Table 3:** Performance of students in evaluation methods used in the Pharmaceutical Care course (N = 30).

	Discursive Written Exam(Mean ± SD)	Seminars(Mean ± SD)	OSCE(Mean ± SD)	Virtual Patient(Mean ± SD)	p-value*
Total assessment score (on a scale of 0 to 10)	7.10 (1.17)	7.68 (1.55)	7.70 (2.01)	9.40 (0.41)	**< 0.01** ^**a**^
**Assessment according to the Likert scale: 1 = Strongly Disagree; 2 = Disagree; 3 = Neither Agree Nor Disagree; 4 = Agree; 5 = Strongly Agree**					
In this type of method I feel a lot of pressure to perform well	3.57 (1.04)	3.93 (1.08)	4.07 (1.08)	3.13 (1.14)	**< 0.01** ^**b**^
This method is useful in verifying my learning	3.47 (0.94)	3.43 (0.82)	3.73 (1.05)	3.97 (0.81)	**0.04** ^**c**^
The criteria for correctness of the assessments were appropriate	3.27 (1.05)	3.10 (1.06)	3.30 (1.29)	3.60 (1.04)	**0.30**
The assigned note reflects my learning	2.70 (1.15)	2.87 (0.97)	3.00 (1.08)	3.30 (1.06)	**0.17**

*Kruskal-Wallis.

^a^differences between Virtual Patients e other evaluation methods,

^b^differences between Virtual Patients and Seminar and OSCE,

^c^differences between Virtual Patients and Seminar.

Thirty of the 34 students were involved in the pre-test evaluation of their perception competence in pharmaceutical care practice and 32 students completed the post-course evaluation. The mean test scores for the pre-test and post-test are given in Table 4. Initially, pre-test competency scores, as evaluated by the students, were as follows: “ability to listen to patients” (mean 3.67, SD 0.96) and “ability to educate patients about behaviors that promote health, maintain wellness, prevent and control disease” (mean 3.43, SD 0.97). In the post-test these competencies received higher average scores (mean 4.16, SD 0.77 and 4.28, SD 0.58, respectively). Other items also showed high scores: “search evidence-based health information to assist with patient pharmaceutical care” (mean 4.16, SD 0.72) and “counsel patients about how and when to take their medications, the duration of pharmacotherapy, precautions and side effects” (mean 4.16, SD 0.68).

**Table 4:** Results of pre-tests (N = 30) and post-tests (N = 32) for students’ perception of competencies in pharmaceutical care practices.

Each question was prefixed by “with the pharmaceutical care course, I feel able to…”	PretestMean (SD)	PosttestMean (SD)	p-value
1. Conduct a complete anamnesis in a patient	2.03 (1.13)	3.38 (1.01)	<0.001
2. Assess patient records	2.83 (1.02)	3.94 (0.50)	<0.001
3. Measure clinical parameters (e.g., blood pressure, capillary blood glucose)	2.27 (1.20)	4.03 (0.90)	<0.001
4. Identify drug-related problems	2.83 (0.99)	3.94 (0.50)	<0.001
5. Classify drug-related problems	2.60 (1.10)	3.91 (0.47)	<0.001
6. Differentiate real and potential drug-related problems	2.37 (0.96)	3.66 (0.65)	<0.001
7. Prioritize drug-related problems	2.70 (0.92)	3.88 (0.61)	<0.001
8. Develop a pharmacotherapeutic plan for the patient	2.73 (1.11)	3.59 (0.67)	0.002
9. Conduct pharmacotherapeutic interventions	2.63 (1.07)	3.78 (0.66)	<0.001
10. Assign priority levels for pharmacotherapeutic interventions	2.50 (1.01)	3.88 (0.66)	<0.001
11. Monitor drug related problems	2.80 (1.03)	4.00 (0.57)	<0.001
12. Adapt the plan of care to specific needs of patients	2.70 (0.92)	3.81 (0.59)	<0.001
13. Determine when to perform the next counseling and what specific information should be collected	2.67 (0.96)	3.75 (0.72)	<0.001
14. Establish monitoring parameters with patients	2.60 (1.07)	3.91 (0.53)	<0.001
15. Search evidence-based health information to provide patient pharmaceutical care	3.03 (1.10)	4.16 (0.72)	<0.001
16. Promote patient adherence to pharmacotherapy	3.23 (1.17)	4.00 (0.62)	0.001
17. Talk about health issues with patients or their caregivers	3.03 (1.13)	3.94 (0.56)	<0.001
18. Counsel patients about how and when to take their medications, the duration of pharmacotherapy, precautions and side effects	3.40 (1.16)	4.16 (0.68)	0.002
19. Counsel patients on the proper use of products for self-monitoring and self-diagnosis (e.g., blood glucose monitors)	2.40 (1.00)	4.00 (0.72)	<0.001
20. Educate patients about behaviors that promote health, maintain wellness, prevent and control disease	3.43 (0.97)	4.28 (0.58)	0.001
21. Communicate with patients or their caregivers	3.33 (1.12)	3.97 (0.90)	0.004
22. Communicate with other health professionals	3.07 (1.05)	3.84 (0.72)	0.002
23. Detect nonverbal signals during patient care	2.70 (0.92)	3.31 (0.82)	0.001
24. Listen to patients	3.67 (0.96)	4.16 (0.77)	0.009
25. Document completely methods of pharmaceutical care practice	2.80 (1.16)	3.75 (0.62)	<0.001

Likert scale: 1 = “Strongly Disagree”; 2 = “Disagree”; 3 = “Neither Agree Nor Disagree”; 4 = “Agree”; 5 = “Strongly Agree.”

In the pre-test, the worst scores were obtained for the competencies “conduct a complete anamnesis in a patient” (mean 2.03, SD 1.13) and “measure clinical parameters” (mean 2.27, SD 1.20). For the post-test, the competencies with the lowest scores were “detect nonverbal signals during patient care” (mean 3.31, SD 0.82) and “conduct a complete anamnesis in a patient” (mean 3.38, SD 1.01). Comparison of pre- and post-test scores revealed statistically significant improvement (*p* < 0.05) for all evaluated competencies.

The students’ assessments of the pharmaceutical care course, its teacher, and their self-assessments were completed by 30 students. The results from the student survey are summarized in Table 5. In their evaluations of the course, 100% of the students indicated strong agreement or agreement that “throughout the course, feedback was provided to help direct learning.” Furthermore 96.67% of respondents strongly agreed or agreed that “the lessons encouraged student discussions in the classroom” and “the lessons had relevance for my professional/personal development.”

**Table 5:** Assessment of Pharmaceutical Care discipline, of teacher and student self-assessment (N = 30).

	1		2		3		4		5		Mean (SD)
ASSESSMENT OF THE PHARMACEUTICAL CARE LESSONS	n	%	n	%	n	%	n	%	n	%	
The lessons stimulated discussion in the classroom.	0	0	0	0	1	3.33	20	66.67	9	30.00	4.27 (0.52)
The lessons stimulated individual study.	0	0	0	0	3	10.00	24	80.00	3	10.00	4.00 (0.45)
The time available for the lessons was appropriate.	0	0	1	3.33	3	10.00	20	66.67	6	20.00	4.03 (0.67)
The learning objectives were explained.	0	0	0	0	3	10.00	18	60.00	9	30.00	4.20 (0.61)
The learning objectives were achieved.	0	0	1	3.33	3	10.00	23	76.67	3	10.00	3.93 (0.58)
During the course, students were provided with opportunities to demonstrate their knowledge.	0	0	0	0	2	6.67	16	53.33	12	40.00	4.33 (0.61)
Throughout the course, feedback was provided to help direct learning.	0	0	0	0	0	0	19	63.33	11	36.67	4.37 (0.49)
The facilities used for the lessons were appropriate.	0	0	2	6.67	5	16.67	16	53.33	7	23.33	3.93 (0.83)
The lessons had relevance for my professional/personal development.	0	0	0	0	1	3.33	22	73.33	7	23.33	4.20 (0.48)
This course is related to other courses of the program.	0	0	3	10.00	2	6.67	14	46.67	11	36.67	4.10 (0.92)
The plan of the course was organized.	0	0	1	3.33	3	10.00	20	66.67	6	20.00	4.03 (0.67)
The students were referred to relevant texts and other study materials	0	0	1	3.33	3	10.00	23	76.67	3	10.00	3.93 (0.58)
**ASSESSMENT OF THE TEACHER**	n	%	n	%	n	%	n	%	n	%	Mean (SD)
Was accessible and available to answer students’ questions.	0	0	1	3.33	0	0	16	53.33	13	43.33	4.37 (0.67)
Encouraged students to participate in discussions.	0	0	0	0	3	10.00	18	60.00	9	30.00	4.20 (0.61)
Encouraged student participation in the practical classes.	0	0	0	0	2	6.67	17	56.67	11	36.67	4.30 (0.60)
Encouraged students to search for evidence and justify their recommendations.	0	0	0	0	3	10.00	20	66.67	8	26.67	4.20 (0.55)
Demonstrated the ability to criticize and receive criticism.	0	0	2	6.67	9	30.00	15	50.00	4	13.33	3.70 (0.79)
Presented the content of the lessons clearly.	0	0	0	0	3	10.00	19	63.33	8	26.67	4.17 (0.59)
Used satisfactory teaching procedures.	0	0	0	0	4	13.33	22	73.33	4	13.33	4.00 (0.53)
Helped clarify the purpose of the course evaluation activities.	0	0	0	0	7	23.33	16	53.33	6	20.00	3.93 (0.69)
Contributed to a favorable learning environment.	0	0	0	0	0	0	25	83.33	5	16.67	4.17 (0.38)
Made good use of class time.	0	0	0	0	0	0	23	76.67	7	23.33	4.23 (0.43)
**STUDENT SELF- ASSESSMENT**	n	%	n	%	n	%	n	%	n	%	Mean (SD)
I felt that I was able to learn the content.	0	0	1	3.33	0	0	21	70.00	8	26.67	4.20 (0.61)
I had difficulty retaining the content that was taught in the class.	6	20.00	14	46.67	8	26.67	2	6.67	0	0	2.20 (0.85)
I had difficulty in visualizing how the content could be applied in practice.	7	23.33	12	40.00	2	6.67	7	23.33	2	6.67	2.50 (1.28)
This course provided me with the opportunity to practice and improve my competencies.	0	0	0	0	3	10.00	18	60.00	9	30.00	4.20 (0.61)
I was motivated to learn more.	0	0	0	0	9	30.00	19	63.33	2	6.67	3.77 (0.57)
As a student I contributed to a favorable learning environment.	0	0	0	0	9	30.00	14	46.67	7	23.33	3.93 (0.74)
The group contributed to a favorable learning environment.	0	0	1	3.33	4	13.33	20	66.67	5	16.67	3.97 (0.67)

(1 = Strongly Disagree; 2 = Disagree; 3 = Neither Agree Nor Disagree; 4 = Agree; 5 = Strongly Agree)

In the student's evaluation of the teacher, 100% of the students indicated strong agreement or agreement that the teacher “contributed to a favorable learning environment” and “made good use of class time.” With regard to the students’ self-assessments, 96.67% of the students strongly agreed or agreed that “they had been able to learn the course content”, and 86.5% strongly agreed or agreed that “the group contributed to a favorable learning environment.” Additionally, twenty-seven students (90%) affirmed that they preferred the pharmaceutical care course using active learning methods to a traditional method, and 96.67% of respondents preferred to use active learning methodologies in other disciplines, indicating that the students were comfortable with learning in a self-directed manner.

## Discussion

In recent years, expanded roles for pharmacists, as providers of health-care services, are increasingly recognized and valued. Moreover, traditional pharmacy education has failed to systematically link practice competencies to patient and population health needs [32]. Thus, preparing pharmacy students for practice in the modern healthcare system requires that we rethink pharmacy teaching methodology and go beyond the traditional lecture-based delivery of factual material, to incorporate those methods that facilitate the effective application of knowledge and development of problem solving skills [33]. Teaching methods should be designed to instruct students in how to provide pharmaceutical care, with a process that evaluates students’ ability to provide this care [27].

According to the International Pharmaceutical Federation (FIP), the capacity to improve therapeutic outcomes, patients’ quality of life, scientific advancement, and public health imperatives are dependent on a foundation of competence [34]. In this context, the literature suggests that pharmacy education is important for students to practice and develop proficiency in the skills, knowledge, and attitudes relevant to achieving the desired performance [35]. In the case of pharmaceutical care, these competencies include the ability to communicate with patients; discuss with patients the appropriate use of medicines; identify, prioritize, and resolve drug-related problems; assess and diagnose based on objective and subjective measures; ensure appropriate medicine administration; provide continuity of care by monitoring patients’ progress through follow-up care and document any interventions [8,34].

In this study a significant improvement in the competencies of students was observed after a pharmaceutical care course that used active learning methods. Several studies have shown that incorporating active learning methods into pharmaceutical care courses can produce positive results in student’s competencies [24, 36–39]. Reid et al. [35] evaluated graduating students' perception of their preparedness to perform pharmaceutical care practice. Students felt that they were more prepared to perform technical pharmaceutical care activities, interact and communicate with patients, and perform administrative tasks as compared to when they began their educational experience.

Bulatova et al. [24] demonstrated that a pharmaceutical care course increased significantly students’ competencies and showed that most students achieved the learning objectives which included: assessing the medical literature relevant to the patient and his or her medications; identify, analyze, resolve and prevent drug-therapy problems; provide patients’ education; and propose an appropriate pharmacist care plan. Darbishire et al. [37] conducted a study using a pre-test and post-test methodology to evaluate students’ confidence in their diabetes care skills, after incorporating active learning techniques through a series of transitional experiences. The results showed that the activities appeared to be effective, since the students’ knowledge increased in areas that were specific to diabetes medication therapy management services. Hudgens and Chirico [38] evaluated student achievement for similar competencies and noted that 70% of the students rated their learning as good or excellent for objectives, classifying drug-therapy problems, data collection and evaluation, assigning priority levels, creating compound goals, and providing patient education.

According to Cipolle et al. [27], the three major steps in the patient care process are the assessment of the patient, his/her medical problems, and any drug therapy problems that have occurred; development of a care plan; and conducting a follow-up evaluation. The competencies evaluated by students as improved after a pharmaceutical care course were: listen to patients, search for evidence-based health information to assist with patient pharmaceutical care, counsel patients about how and when to take their medications, determine the appropriate duration of pharmacotherapy, elucidate precautions and side effects and educate patients about behaviors that promote health, maintain wellness, prevent and control disease. Although not the main steps of pharmaceutical care, these competencies are fundamental to many pharmaceutical clinical services and assist in achieving the outcomes of patients. Furthermore, the finding that some competencies were better learned than others allows curriculum planners to focus on areas where more learning opportunities are required.

Surveys that capture the perceptions and opinions of students are commonly used in health science research [40, 41]. However, the literature suggests that students sometimes over estimate their skills while they still are in the didactic phase of their education. Exposure to real pharmacy practice gives them a sense of their true abilities and skills [37, 41, 42]. This awareness is a positive step forward in their professional development. So, in pharmacy education it is interesting to combine self-evaluations with other objective ways of measuring student learning, e.g., tests in class, pre-/post-course tests, formative vs. summative assessments, computer-assisted instruction, etc [42,43].

In the pharmaceutical care course that is the subject of this report, the teachers wanted to provide students with multiple methods of assessment in class, according to the learning objectives, and to increase the number of opportunities students had to demonstrate application of learning. The tests for the evaluation course were carefully developed so that students were required to use the information to demonstrate mastery of specific course objectives rather than simply recalling memorized facts. Hence the use of a discursive written exam with use of video for resolution of problem situations, seminars with clinical cases, OSCE with evaluation of student performance facing a simulated situation, and virtual patients.

The results of these assessment methods are in accord with results of previous studies on the advantages of active methods in learning [44–46]. Stewart et al. [47] affirmed that, ideally, all educators would engage in pedagogical strategies that demonstrate superior student learning and satisfaction. Assessment of student learning outcomes is a high priority for colleges of pharmacy and is mandated by the Accreditation Council for Pharmacy Education (ACPE) as essential for quality improvement of the curriculum [16–18]. According to the literature, assessment plays a key role in the learner-centered teaching approach, and must be focused on specific, desired learning outcomes. That is, the curriculum should be planned around student learning outcomes that link knowledge, skills, behaviors, attitudes, and values [16,48].

In evaluation the methods applied for the current study, the best assessment method for students was the virtual patient. Additionally, the use of this technology was noted as one of the strengths of the course by students. As active learning techniques have been encouraged in pharmacy education, an increase in the use of technology has often followed [47]. The virtual patient technique is especially useful for training students for pharmaceutical care practice because it enables the application in clinical practice of the theoretical knowledge acquired by the students during their studies [49]. A further relevant issue is the application of knowledge in pharmacy practice. Although traditional testing may form a component of assessment in a learner-centered course, assessments should also represent how course content will actually be used in practice [50].

Overall, students were positive in their evaluations of instructional content and methods of the course. Most students agreed that the pharmaceutical care course would help them apply skills in actual pharmaceutical practice, and they affirmed that they preferred a course using active learning methods rather than traditional methods. Similar results are found in literature [33,37]. One example is the study of Van Amburgh et al. [33], which found that students perceived the merits of using active learning as better retention of material and improved application of knowledge and critical thinking (29%). Barclay [51] reported that this ability to identify students with a specific learning predilection allows educators to provide a more individualized teaching approach.

However, the course also presented some limitations as the little contact with real patients. According to Hudgens and Chirico [38], students should be exposed to practice environments that will allow learning experience within the context of actual pharmacy practice, which will help empower them to practice in covenant relationships with patients. Practice experience with patients has a positive impact on pharmacy education, facilitating early professional socialization. It enables students to develop their professional attitudes and skills, providing them with timely clinical exposure as the profession takes on a more patient-focused approach [52]. In the case of pharmaceutical care, these relationships with patients can promote communication skills in the students, facilitate the identification actual or potential drug-related problems, and encourage students to engage in informed shared decision-making with patients.

In this study, in view of the limited availability of actual patients to develop pharmaceutical care practice, a set of active learning techniques were used that can decrease the gap between training and actual patient experience. The literature notes that the laboratory setting is ideal for providing students with hands-on experience in a low-risk environment, by instilling both confidence and competence in students [37]. The FIP supports the teaching of practical pharmacotherapy and pharmaceutical care issues in a controlled setting [34]. Thus, real patient cases that pertained to disease states covered in the current course were used, providing relatively realistic situations. Further, simulations were used to encourage students to address motivational and behavioral issues of patients.

Many health care educators have described the use of simulation as a successful teaching strategy [53–56]. Simulation is considered a valuable teaching technique that has been integrated in pharmacy education to prepare students for pharmacy practice [57]. Simulated experiences provide students the opportunity to engage in critical thinking and clinical decision making [58]. In pharmacy education, simulation has been used successfully in areas such as therapeutics, communication, physical assessment, patient safety, and interprofessional health care team skills [57]. Therefore, when students’ understanding of pharmaceutical care starts to take shape, simulated case scenarios can be used to develop pharmaceutical care competences, such as development of an empathetic attitude [39].

It is noteworthy that all experiences used in a pharmaceutical care course should have well defined outcomes that lead to the primary aim of optimizing the educational experience for the student [59]. To achieve the improvement of quality, a course review process should focus on foundational aspects of teaching, learning, and assessment, such as the presence of appropriate learning objectives, and activities and assessment methods consistent with learning objectives [21]. Development of student learning outcomes is the foundation for building curricula because learning outcomes must guide content development and the selection of instructional methodologies. In addition, to enhance the educational process, teachers should guide student learning. Teachers should engage students in active learning that allows for “construction” of their own knowledge, provide students with sufficient time in the curriculum to reflect upon and learn from new experiences, integrate knowledge and concepts rather than teach them in isolation, and use a variety of learning approaches in their courses. This prepares students to be the change agents needed to enhance the quality of direct patient care provided by pharmacists [39].

The present study is not without limitations. First, limitations inherent in this method include differences in personal factors among the participants. Second, since the active learning approach was not compared to a traditional teaching methodology, it cannot be determined whether the former is the superior approach for the teaching of pharmaceutical care. Furthermore, the active learning strategies were not test individually, which prevents to evaluate the importance of each in the learning process.

## Conclusion

By incorporating active learning methods in a Brazilian pharmaceutical care course, students demonstrated significant improvements in their competencies and expressed satisfaction with the course. Although the results are limited to the study population, it appears that an active learning course with a combination of several teaching and evaluation methods can enhance learning pharmaceutical care competencies. Future studies should compare active learning to traditional methods.
